# What does a consent conversation for whole genome sequencing look like in the NHS Genomic Medicine Service? An observational study

**DOI:** 10.1038/s41431-024-01749-x

**Published:** 2024-11-26

**Authors:** Holly Ellard, Huda Alfardus, Saskia Sanderson, Celine Lewis

**Affiliations:** 1 Population, Policy and Practice Department, UCL Great Ormond Street Institute of Child Health, London, UK; 2 Institute of Psychiatry, Psychology and Neuroscience, King’s College London, London, UK; 3 Department of Behavioural Science and Health, University College London, London, UK; 4 UK Office for Life Sciences (OLS) / NIHR Mental Health Mission, London, UK; 5 Public Health Genomics (PHG) Foundation, Cambridge, UK; 6 NHS North Thames Genomic Laboratory Hub, Great Ormond Street Hospital for Children NHS Foundation Trust, London, UK

**Keywords:** Genetic testing, Genetic testing

## Abstract

Patient choice consent for whole genome sequencing (WGS) through the Genomic Medicine Service in England covers consent to diagnostic testing and an invitation to the National Genomic Research Library (NGRL). Little is known about what consent conversations for WGS look like in practice. We audio-recorded and analysed the content and structure of consent appointments (*n* = 26) between healthcare professionals (HCPs) and parents of children with rare disease across seven NHS Trusts. Appointments frequently covered the potential findings from testing, implications for family members, and DNA storage, but often omitted that data may be reanalysed in the future if a diagnosis is not made. Consent to the NGRL was typically sought during the same appointment; these discussions varied in content, but frequently included a background to the NGRL and data security. HCPs often tempered expectations around what WGS can achieve and asked questions to clarify parents’ understanding, but less commonly elicited parents’ values and concerns. Administrative tasks were time-consuming, but took less time when consent was recorded digitally. Future training should emphasise how to elicit patients’ values and concerns. Digital infrastructure and hiring roles such as genomic associates to support consent may be important strategies to meet the workload demands of WGS.

## Introduction

Whole genome sequencing (WGS) is now being used in the NHS in England as a diagnostic test for some rare diseases through the Genomic Medicine Service (GMS). This approach sequences a patient’s entire genome but restricts analysis to variants within a subset of genes with a known link to their phenotype, which can include variants within hundreds of genes. This technology and process has a diagnostic yield of 25% among NHS rare disease patients, increasing to 40–55% for some conditions [1]. Other possible outcomes from WGS are: variants of uncertain significance (variants that do not have sufficient or clear evidence to ascertain pathogenicity and may require further action to resolve); uninformative results (when no relevant or pathogenic variation is identified); and incidental findings (results that are unrelated to the reason for testing, such as an increased risk of a future health condition). Even when a diagnosis is made, uncertainties may remain around prognosis and impact on care. The complexity of WGS and multiple possible outcomes and implications, as well as potentially inflated expectations about the utility of WGS [2, 3], can make fully informed consent challenging [4].

In the GMS, consent follows a new ‘patient choice’ model, which covers not only consent to diagnostic testing, but also an offer for patients to contribute their data to the National Genomic Research Library (NGRL), a database which approved researchers can access for approved research purposes [5]. Consent to the genomic test and the NGRL is captured on a standard Record of Discussion (RoD) form [6]. A key feature of the GMS is that specialists outside of clinical genetics can order WGS, encouraging the ‘mainstreaming’ of genomics [7]. To ensure that professionals from all clinical backgrounds have the knowledge, skills and behaviours necessary to facilitate consent to WGS, a cross-professional competency framework has been developed [5]. Additionally, resources to support the consent conversation include a guide for requesting WGS for rare disease [8], online learning courses for professionals [9], and patient information leaflets [10].

To our knowledge, no study has investigated the conversation that takes place between healthcare professionals (HCPs) and patients during the consent process for WGS. Insight can be gleaned from work conducted during the 100,000 Genomes Project (100 kGP) – a hybrid clinical-research project that sequenced 100,000 genomes from NHS patients/ their relatives to pilot the introduction of WGS into clinical practice. In an observation study of 21 consent appointments for the 100 kGP [11], it was found that appointments largely mirrored the written consent form, with most covering the potential benefit of WGS for the patient (20/21), implications for relatives (19/21) and that results may not provide a diagnosis (17/21). In interview studies [12–14], participants tended to recount the 100 kGP consent process as a positive experience. However, some participants were found to have poor understanding of the information provided during the consent process [12–15]; made consent decisions based upon trust, opposed to a sound understanding of the project [13, 14]; and had unmet expectations from taking part [13, 16], raising questions about the overall effectiveness of the consent process.

Whilst guidance and resources have been developed to support HCPs to facilitate consent to WGS through the GMS, how these translate into consent conversations has yet to be explored. Observations of these conversations could help identify topics that are challenging to discuss or frequently omitted and provide insights for HCPs routinely conducting these conversations. Moreover, observations could help identify differences across the types of HCPs (e.g. consultants or genetic counsellors) delivering these conversations, and their speciality (e.g. clinical genetics or another speciality). We analysed audio-recorded consent appointments for paediatric patients offered WGS for rare disease diagnosis, with the aims to: describe the overarching structure of appointments in terms of length and content; identify any differences according to which setting appointments took place and who conducted it; and identify what questions parents/patients ask.

## Methods

### Methodological approach

We audio-recorded, transcribed and qualitatively and quantitatively analysed the content and structure of consent conversations between HCPs and parents of children being offered WGS for rare disease diagnosis.

### Participants and recruitment

Participants were HCPs conducting consent conversations and parents of paediatric patients offered WGS through the GMS across seven regionally diverse NHS Trusts. We aimed for each Trust to identify three or four consent appointments for observation. Key contacts at each Trust were approached to identify HCPs consenting patients to WGS for this study, ranging in both speciality (genetic and mainstream specialists) and professional role. Those HCPs were provided with further information about the study and invited to participate via email. HCPs who were interested in taking part were asked to complete a consent form and participant demographic form. To identify suitable appointments for observation, HCPs were asked to notify the study team of upcoming WGS consent appointments for parents of paediatric patients. Parents were eligible if they were parents of a child with an undiagnosed rare condition (<16 years of age); had capacity; were able to read information materials; and were having the discussion in English or with a professional translator present. Appointments were included in this study regardless of whether consent for WGS was given, declined, or deferred.

To enrol parents, HCPs or a member of their staff approached parents ahead of the appointment to explain the study, provide them with a participant information sheet, and assess interest in participation. Alternatively, parents were approached by C.L. in the waiting area prior to their appointment with information about the study and an offer to participate. Parents who were interested in participating were asked to complete a consent form and participant demographic form before the appointment began. Young people who had capacity to assent to take part were given an age-appropriate participant information sheet and asked to sign an assent form.

Data collection took place between November 2021 and October 2022. C.L. attended and audio-recorded all consent appointments. Field notes were taken during the appointment to gather supplementary data, such as testing indication, sampling processes, and administrative tasks.

### Data analysis

Audio-recordings were transcribed verbatim by an external transcriber. Deductive content analysis [17] using a codebook approach was used to characterise the content of appointments. The codebook was adapted from that developed by C.L. and colleagues in an observational study of consent appointments for the 100 kGP [11]. Minor amendments to the codebook were made to account for differences between the 100 kGP and GMS (e.g. that consenting to the NGRL was optional). Three transcripts were coded by H.A. and C.L. Codes were compared, and discrepancies were resolved. H.A. coded the remaining transcripts independently. During this analysis phase, questions parents asked were identified and thematised, and consenters’ questions were identified and coded as either “open-ended” (which prompts the responder to provide feedback in their own words) or “closed-ended” (which prompts the responder to provide a ’yes’ or ‘no’ style answer). H.E. independently coded the theme of psychosocial aspects of the consent conversation within the codebook. To explore the structure of appointments, the number of words pertaining to a theme were counted (C.L.) and the time spent on key components of the conversation was measured (C.L. and H.E.).

## Results

Twenty-eight families were invited to participate, of whom two declined, giving a total of 26 consent appointments observed across seven NHS Trusts in England (Table 1). The characteristics of participating HCPs and parents are detailed in Tables 2 and 3, respectively.

**Table 1:** Details of whole genome sequencing consent appointments.

Characteristic		Frequency (*n* = 26)	Percentage of total appointments
Role of healthcare professional delivering consent appointment	Consultant	12	46%
	Speciality Doctor	4	15%
	Specialist Registrar	1	4%
	Clinical Fellow	2	7%
	Pre-registration Genetic Counsellor	1	4%
	Genomic Associate	6	23%
Delivery mode of appointment	Face-to-face	18	69%
	Video call	4	15%
	Telephone	4	15%
Testing indication	R29 Intellectual disability	15	58%
	R27 Paediatric disorders	9	35%
	R59 Early onset or syndromic epilepsy	3	12%
	R61 Childhood onset hereditary spastic paraplegia	2	8%
	R77 Hereditary neuropathy - PMP22 copy number	1	4%
	R78 Hereditary neuropathy or pain disorder	1	4%
	R87 Cerebral malformation	1	4%
	R89 Ultra-rare and atypical monogenic disorders	1	4%
	R54 Hereditary ataxia with onset in adulthood	1	4%
	R84 Cerebellar anomalies	1	4%
	R135 Paediatric or syndromic cardiomyopathy	1	4%

**Table 2:** Characteristics of healthcare professionals participating in an observational study of whole genome sequencing consent appointments.

Characteristic		Frequency (*n* = 20)	Percentage of total sample
Age (mean 39 years, range 22 years - 54 years)	18–35	6	30%
	36–50	9	45%
	≥50	2	10%
	Missing	3	15%
Gender	Female	16	80%
	Male	4	20%
Professional background	Clinical genetics	14	70%
	Paediatrics	1	5%
	Paediatric Neurology	2	10%
	Neurology	1	5%
	Biomedical science or biochemistry	2	10%
Current role	Consultant	11	55%
	Specialist Registrar	1	5%
	Speciality Doctor	1	5%
	Clinical fellow	2	10%
	Pre-registration Genetic Counsellor	1	5%
	Genomic associate	4	20%
Years in role (mean 8 years, range <1–16 years)	<1 year	7	35%
	2–5 years	5	25%
	6–10 years	2	10%
	>10 years	4	20%
	Missing	2	10%
Involved in consenting for 100,000 Genomes Project	Yes	12	60%
	No	8	40%
Experience with genomics	None	1	5%
	Some	6	30%
	Lots	13	65%

**Table 3:** Characteristics of parents participating in an observational study of whole genome sequencing consent appointments.

Characteristic		Frequency (*n* = 42)	Percentage of total sample
Age (mean 38 years, range 19–56 years)	18–35	12	29%
	36–50	24	57%
	≥50	1	2%
	Missing	4	10%
Gender	Female	25	60%
	Male	17	40%
Education	Master’s degree	2	5%
	Undergraduate degree	9	21%
	Vocational qualification	2	5%
	A Level	4	10%
	GCSE	15	36%
	None	5	12%
	Missing	5	12%
Ethnicity	White or White Other	28	67%
	Asian	14	33%

### Consent appointment structure

#### HCPs delivering appointments

Appointments were delivered by a consultant (12/26), genomic associate (GA) (6/26), speciality doctor (4/26), clinical fellow (2/26), specialist registrar (1/26), or pre-registration genetic counsellor (1/26). In 19/26 appointments, these HCPs specialised in clinical genetics. In the other 7/26 appointments, HCPs specialised in paediatrics or neurology.

**Fig. 1:**
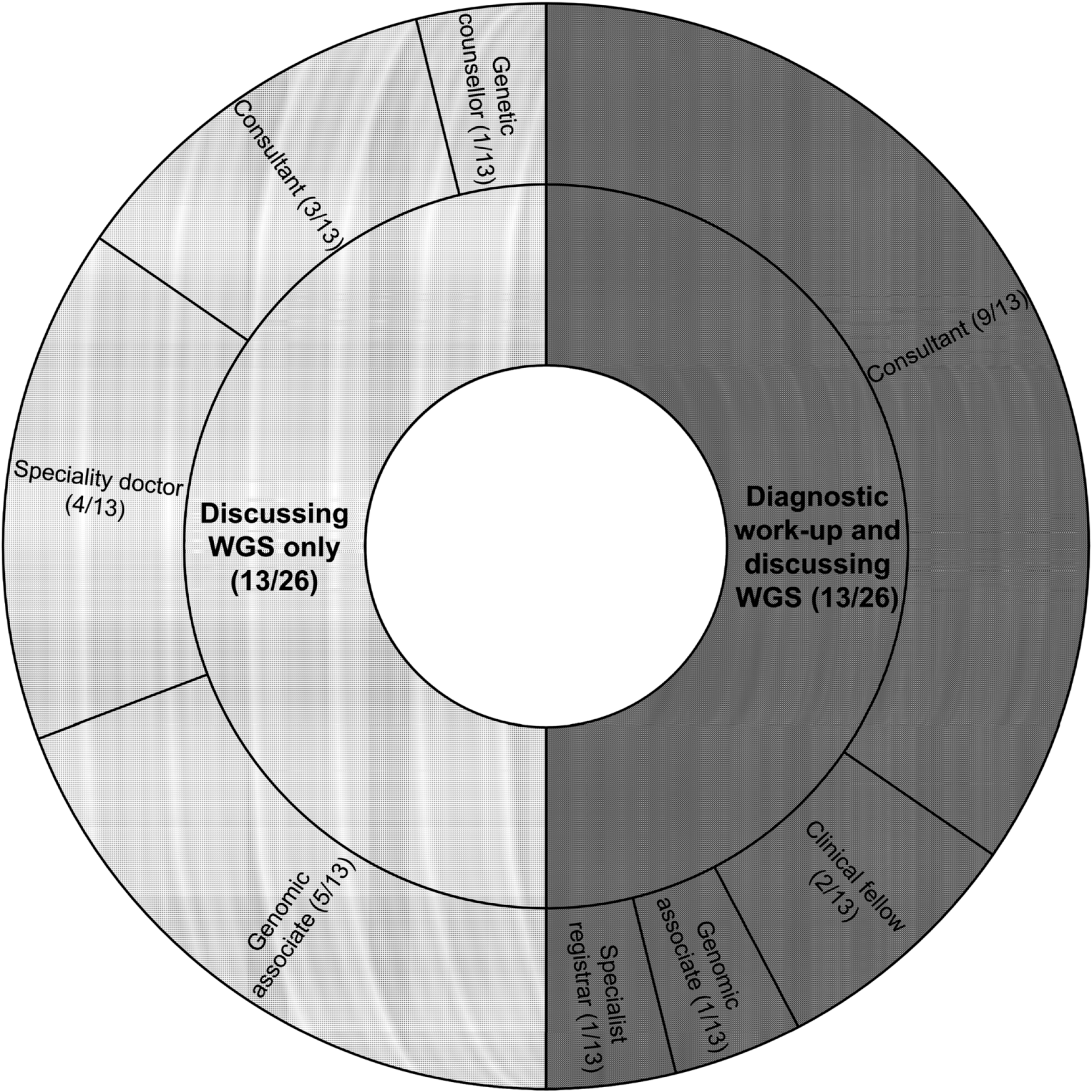
A sunburst diagram showing the task(s) involved in whole genome sequencing (WGS) consent appointments and the type and frequency of healthcare professionals conducting these tasks. The inner circle shows the number of appointments that involved discussing WGS only and the number of appointments that involved diagnostic work-up and discussing WGS. The outer circle shows the type and number of healthcare professionals that led those appointments.

Half of appointments (13/26) involved only discussing and consenting to WGS, whereas the other half also involved diagnostic work-up. The HCPs that led these two appointment types are outlined in Fig. 1.

#### Duration

The consent conversation ranged from 5 min 16 s to 25 min 2 s (mean 13 min 59 s, median 14 min). Appointments delivered by HCPs specialising in clinical genetics took an average of 15 min 16 s, whereas appointments delivered by HCPs from another speciality took an average of 10 min 6 s. Appointments delivered by GAs and pre-registration genetic counsellors tended to be longer (mean 15 min 57 s) than appointments delivered by doctors (mean 13 min 7 s). Conversations took less time in appointments that only involved consenting to WGS (mean 12 min 26 s), compared to appointments including diagnostic work-up (mean 15 min 34 s).

Completing and signing the RoD (the form to record consent, covering family and wider implications, uncertainty, unexpected information, DNA storage, health records, and research) took an average of 4 min 46 s (range 19 s–24 min 33 s). In appointments where consent was recorded digitally (11/26), administrative tasks took an average of 1 minute 54 s, compared to 7 min 5 s where parents provided a ‘wet ink’ signature (13/26).

Ahead of the appointment, 7/26 families were sent an information leaflet about WGS, and 6/26 were sent the RoD.

#### Content

Discussing biological concepts within genetics took up 15% (range 5–32%) of the appointment on average; the possible main findings from WGS took up 29% on average (range 15–68%); and the possibility of incidental findings took up 17% on average (range 6–33%). In all but two consent appointments families were invited to the NGRL, taking up 29% (range 11–61%), on average, of the appointment.

GAs and pre-registration genetic counsellors covered an average of 69% of all topics, while doctors covered an average of 51%. HCPs specialising in clinical genetics covered an average of 57% of topics, compared to 51% covered by HCPs from another speciality.

### Consent to diagnostic testing

**Fig. 2:**
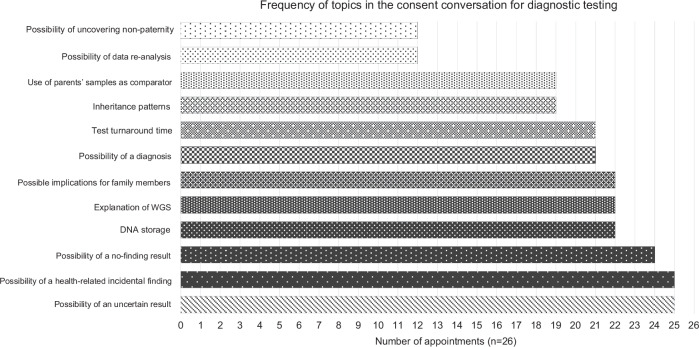
A bar chart displaying the frequency of topics discussed during consent conversations for diagnostic whole genome sequencing (WGS) in the NHS Genomic Medicine Service. Key topics covered during the consent conversation are detailed on the vertical axis, with the number of appointments covering those topics detailed on the horizontal axis.

The frequency of topics covered during consent conversations for diagnostic testing are summarised in Fig. 2. Commonly discussed topics included the possibilities of an uncertain result (25/26), health-related incidental finding (25/26), or not receiving a diagnosis (24/26) (quote below), and results having implications for relatives (Table 4: Q1) (22/26).

*“It’s also possible that we do the test and we may not find anything significant other than what we’ve previously found and that can be difficult because it doesn’t always completely rule out there being a genetic cause”* Observation 16 (GA, Clinical Genetics)

**Table 4:** Illustrative quotes from content analysis of consent appointments for whole genome sequencing in the NHS Genomic Medicine Service.

	Element of discussion	Identifier	Illustrative quote
Q1	Consent to diagnostic testing	Observation 16	“There is a chance that other members of your extended family as well could be carrying that genetic change and so obviously if there was anyone in the family with similar features to X or anyone that was concerned or worried then we could provide some more information…”
Q2	Consent to NGRL participation	Observation 11	“It’s not available for insurance companies or anyone outside health research really”
Q3	Psychosocial aspects (diagnostic testing)	Observation 4	“We want to know why she’s like she is, don’t we?“
Q4	Psychosocial aspects (NGRL)	Observation 16	“I’m happy for my data to be used because I think it’s really important that the research is able to be carried out so obviously when parents have children with illnesses or unknown conditions, because obviously we as parents always look for answers and obviously if the medical team can’t do the research and investigate, we as parents won’t be able to get our answers”
Q5	Psychosocial aspects (diagnostic testing)	Observation 22	“It’s hard to get extra support, like speech and language, extra help in school. If you have a diagnosis it’s easier.”
Q6	Psychosocial aspects (NGRL)	Observation 2	“the benefit I guess we could say would be that if we don’t find answer yet for X, if she’s in that research element…”
Q7	Psychosocial aspects (diagnostic testing)	Observation 26	“My fear is we’re going to learn something… that he will develop when he’s 40 or 50… we’d hate to know that”
Q8	Psychosocial aspects (diagnostic testing)	Observation 6	“We very often don’t find anything, so it’s worth trying, but we might not have an answer for you at the end of it”
Q9	Parents’ questions (diagnsotic testing)	Observation 21	“Can the school use that to apply for extra time for him, for his tests, with him being under investigation?”
Q10	Parents’ questions (NGRL)	Observation 17	“Is that quite an open thing?“
Q11	Engaging parents in discussion	Observation 25	“So what questions do you have with regards to that?”

Topics covered less frequently included that data may be reanalysed in the future (12/26) (quote below) and the risk of uncovering non-paternity (12/26).

*“The good thing with this test is that actually you have the data forever and you can always go back and interrogate the data as more knowledge accumulates about new genes or old genes that we learn about new types of presentation”* Observation 1 (Consultant, Paediatric Neurology)

### Invitation to the NGRL

**Fig. 3:**
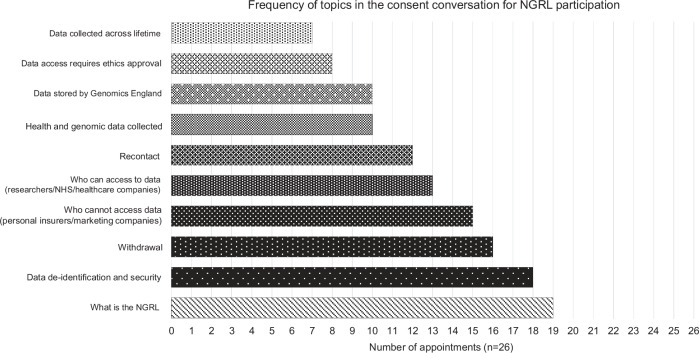
A bar chart displaying the frequency of topics discussed during consent conversations for participation in the National Genomic Research Library (NGRL) in the NHS Genomic Medicine Service. Key topics covered during the consent conversation are detailed on the vertical axis, with the number of appointments covering those topics detailed on the horizontal axis.

The frequency of topics covered about the NGRL are summarised in Fig. 3. Over half of appointments covered contextual information about the NGRL (19/26); data de-identification and storage (18/26); withdrawal (16/26); and that data cannot be accessed by personal insurers or marketing companies (15/25) (Table 4: Q2). Aspects less frequently covered included that: families can be recontacted (12/26) (quote below); data collection includes genomic and health data (10/26), stored by Genomics England (10/26); data access requires approval by a research ethics committee (8/26); and research can take place across the patient’s lifetime (7/26).

*“We will recontact you, either us or the genetics team or even Genomics England with us as your clinical team, to contact you if we do find something, or especially if there’s any research that might be beneficial to you”* Observation 14 (Consultant, Paediatrics)

### Psychosocial components

#### Clarifying goals

Goals that parents hoped to achieve through diagnostic testing included to improve their understanding of their child’s condition (3/26) (Table 4: Q3), duty to their child (2/26), to improve others’ understanding of their child’s condition (1/26), relief from excluding a genetic condition (1/26), and a diagnosis for better school support (1/26), exemplified with:

*“If we don’t get a diagnosis and we don’t know how to support him, they’ll just have him as an under-achiever”* Observation 21 (Parent)

Parents’ goals relevant to NGRL participation included helping others (4/26) (Table 4: Q4) and the search for answers (2/26), for example:

*“Like it said… two years down the line that information could be used to find something”* Observation 21 (Parent)

HCPs’ personal reflections around the value of diagnostic testing included for families to receive an answer and better understand their child’s condition (7/26) (quote below); the opportunity for reanalysis (4/26); to inform prognosis (4/26); to avoid the need for further tests (2/26); to provide information for relatives (2/26); to improve school support through a diagnosis (2/26) (Table 4: Q5); and the value of knowing all testing avenues have been explored (2/26).

*“…For a lot of families just being able to explain ‘ah, this is why it’s happened’…”* Observation 11 (Speciality doctor, Neurology)

HCPs’ reflections on the value of NGRL participation included a further opportunity to find answers (6/26) (Table 4: Q6); contributing improved knowledge of conditions and treatments (4/26) (quote below); the opportunity to reanalyse data (1/26); and contributing improved knowledge of genetic changes (1/26).

*“The potential benefits of being part of the research library is it can help us to learn more about genetic conditions and help people in the future”* Observation 20 (GA, Clinical Genetics)

#### Parents’ concerns

Concerns raised by parents included fear of incidental findings (1/26) (Table 4: Q7), distress for their child from further blood sampling (1/26), and fear of receiving a difficult prognosis (1/26), demonstrated with:

*“We would like to get tested but we’re just afraid of what we might find… I just don’t want to find anything that will shorten his life, I don’t want to know his life on a piece of paper”* Observation 26 (Parent)

#### Managing expectations

HCPs sought to manage parents’ expectations around the likelihood of receiving a diagnosis (26/26) (Table 4: Q8), the potential of diagnosing a genetic condition that can’t be treated (6/26) and diagnosing a genetic condition that does not inform prognosis (4/26), for example:

*“Sometimes these conditions are very rare, so sometimes even if we find the cause it’s difficult to predict what will happen”* Observation 22 (Consultant, Clinical Genetics)

### Parents’ questions

Parents’ questions about diagnostic testing included: logistical questions around sampling (7/26) and the testing process (7/26); what unexpected results the test could generate (3/26) (quote below); the scope of the test (2/26); how WGS differs from the deciphering developmental disorders study (1/26); the success rate of the test (1/26); whether testing could facilitate additional school support (1/26) (Table 4: Q9); and the purpose of testing (1/26).

*“Does it show owt [anything] for Crohn’s or ulcerative colitis?”* Observation 5 (Parent)

Regarding the NGRL, parents queried data de-identification (3/26) (quote below); data access (2/26) (Table 4: Q10); eligibility for participation (1/26); difference from other genomic studies (1/26); and whether participation could impact travel insurance (1/26).

*“How can they contact us if it is held anonymously?”* Observation 8 (Parent)

### Engaging parents in discussion

On average, the proportion of words spoken by parents during discussions of WGS was 11% (range 2–29%). HCPs invited parents to clarify their understanding using closed-ended questions in 25/26 appointments, and open-ended questions in 2/26 appointments (Table 4: Q11). HCPs elicited parents’ views and concerns relevant to the consent decision by asking closed-ended questions in 16/26 appointments, and open-ended questions in 6/26 appointments, such as:

*“So how is that all sounding?”* Observation 5 (Consultant, Clinical Genetics)

## Discussion

Consent conversations for WGS in the GMS usually covered the types of main findings, the possibility of incidental findings, that results may have implications for relatives, and DNA storage, but less often data reanalysis. Most appointments sought consent to the NGRL at the same time as diagnostic testing. The length of consent conversations ranged considerably between appointments; administrative tasks were an additional time-consuming element but took less time when consent was recorded digitally. HCPs often invited parents into discussion by asking questions to clarify their understanding or, less commonly, to elicit their views and concerns.

As the number of whole genomes sequenced through the GMS continues to expand, the lack of a suitably trained and available workforce to seek consent has been reported in a previous study as a challenge to early implementation [18]. In that study, interviews with key stakeholders recognised that employing staff such as GAs to support the consent process was a valuable strategy, enabling doctors to concentrate on more specialist tasks. GAs represent a novel role developed specifically to support the provision of WGS through the GMS, with formal guidance on role remit and qualification/training requirements beginning to emerge [19, 20]. In this study, GAs covered more topics than doctors and delivered consent conversations that lasted longer on average. These findings support the place of facilitating consent within the remit of GAs. Given their lower pay band compared to doctors, employing GAs to support the consent process could be a cost-effective way to address workforce challenges in the GMS [18]. That said, the difference in the number of topics covered by GAs compared to doctors could reflect a greater level of familiarity between doctors and their patients compared to GAs, who may be meeting patients/parents for the first time at the consent appointment. For example, doctors may be able to tailor discussion to what they already know about the family and their diagnostic journey, whereas GAs may have an increased reliance on the RoD structure. In interviews with genetic counsellors and research coordinators about their experiences of obtaining consent for WGS [21], experience enabled less emphasis to be placed on standard elements in the consent form and instead focus on addressing misunderstandings. Explicitly elicited patient perspectives would inform which HCPs are considered acceptable to deliver the consent conversation.

In this study, consent conversations took a median of 14 min, with an additional 5 min spent on administrative tasks. This is less than the median length of consent appointments for the 100 kGP (27 min) reported in a previous observational study with a similar design [11]. This difference could reflect that ‘consenters’ (those seeking consent) in that study were mostly (70%) research assistants or co-ordinators, whereas consenters in this study were HCPs. Alternatively, it could indicate that the consent process for WGS has been streamlined since the 100kGP, enabling consent to be obtained in a shorter timeframe. For example, compared to the 100 kGP, patients offered WGS through the GMS no longer have the option to opt-in or opt-out of receiving incidental findings, meaning that time is no longer required to facilitate this decision or complete the associated forms [22]. This study also found that an online platform to record consent improved efficiency, which supports the notion that digital infrastructure is a key enabler for the GMS [18]. In our study, consent conversations delivered by HCPs in non-genetic specialities took the least amount of time and covered fewer topics than appointments delivered by HCPs specialising in clinical genetics. Possible reasons for this could include insufficient time in mainstream clinics or insufficient HCP familiarity with seeking consent to genomic testing in mainstream specialities; research is needed to explore the preparedness and early experiences of mainstream HCPs delivering WGS.

In this study, certain information topics from the RoD were verbally conveyed in nearly all appointments, whereas others were often omitted. This could reflect a move toward ‘appropriately’ (rather than ‘fully’) informed consent for WGS, where information provision is tailored to what an individual would find most helpful to their decision-making [23]. This approach aligns with practices of consent for genomic sequencing described elsewhere in the world [24, 25]. We observed large ranges in the amount of time dedicated to each component of the appointment (e.g. biological concepts, main findings etc), which could suggest that time was apportioned according to what was most important to parents or to areas they needed extra support to understand. However, we do not know the extent to which other factors, such as time pressure, influenced the content and structure of appointments.

**Fig. 4:**
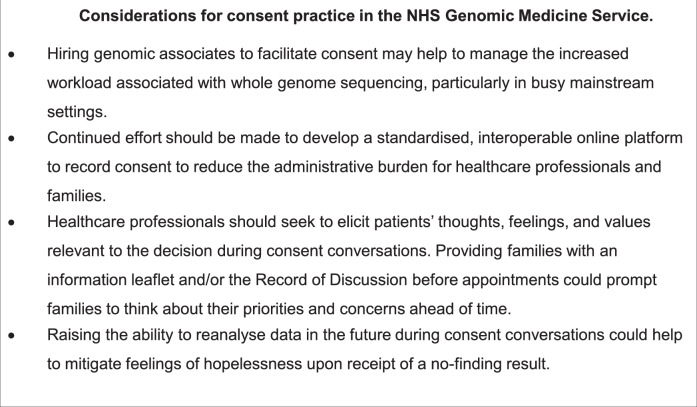
Considerations for consent practice in the NHS Genomic Medicine Service. Reflections on dicussing and taking consent for whole genome sequencing in the NHS Genomic Medicine Service from this observational study.

By understanding what matters to patients, HCPs can share relevant information about the benefits and harms of WGS specific to an individual [26]. However, we found that consent conversations were dominated by information-giving on the part of the HCP with little contribution from parents, and that several HCPs did not ask questions to prompt parents’ views and concerns relevant to the decision. Similarly, in an observation study of 100 kGP consent appointments [11], less than a third of appointments provided opportunities for patients to express their concerns, views, or questions. In a longitudinal survey that assessed decision regret among 100 kGP participants [27], regret was highest among those who had the greatest concerns about WGS at the time of consent; those authors concluded that HCPs delivering WGS must be skilled at eliciting patients’ views and concerns. Training for GMS providers may need to reinforce the importance of asking open-ended questions to prompt perspectives, as well as other counselling and communication techniques to engage patients in discussion (Fig. 4). To supplement existing online training resources on facilitating consent to WGS [28], NHS educational teams could develop continuing professional development workshops to enhance and practise these skills. Additionally, families could be sent an information about WGS before their appointment to consider their questions ahead of time. This happened in some appointments in this study. Other potential barriers to exploring patients’ views could include time constraints and difficulty maintaining attention, which have been reported as challenges to obtaining consent among genetics professionals interviewed about their experiences of delivering WGS [21, 29] Further research is needed to understand if this reflects the experiences of GMS providers.

Several HCPs in this study tempered expectations around the diagnostic yield of WGS during consent conversations, as well as limits to the utility of a genetic diagnosis. In interview studies with clinicians delivering WGS, fostering realistic expectations of what WGS can achieve has been identified as a priority for pre-test counselling [21, 24, 30], further highlighted among studies concluding unmet expectations from genomic testing are linked with negative experiences. For example, in interviews with parents of children that underwent WGS, unmet expectations left many parents with uncertainty, disappointment, and despair [2]. In an interview study with parents who did not get a diagnosis from the 100kGP, many parents were found to experience an emotional rollercoaster including profound disappointment when receiving a no-finding result, and loss of hope left some parents feeling isolated [31]. The authors of that study recommend that data reanalysis should be addressed as part of promoting hope among parents with a no-finding result. This was not always a component of consent conversations in this study, but by making parents aware of the ability to reanalyse data during pre-test counselling, feelings of intense hopelessness experienced at the point of receiving a no-finding result could be mitigated.

### Strengths and limitations

Our content analysis of audio-recorded appointments provided objective, real-world insight into consent conversations taking place in the GMS. Appointments were delivered by a range of HCPs, including GAs and mainstream clinicians. However, most HCPs were involved in consenting to the 100kGP and have ‘lots’ of experience with genomics (Table 2). Our findings cannot be generalised beyond the small sample of appointments observed. Observations took place early on in GMS implementation so do not reflect how communication styles may have developed over time. In addition, factors that may have influenced the dialogue, such as families’ prior interactions with clinical genetics, information given outside of appointments, and genomic literacy, were not assessed.

## Conclusion

Consent conversations covered key information about diagnostic testing and taking part in research, provided opportunities for families to clarify their understanding, and often sought to manage expectations about WGS. Although topics verbally covered did not always encompass every element of the RoD, a one-size-fits-all approach to information provision is not a goal of good practice of consent. Rather, clinical judgement about the specific circumstances of each person and decision to tailor the exchange of information is required. Future work should seek to understand families’ experiences of, and preferences for, the consent process.

## Data Availability

Portions of de-identified transcripts are available upon reasonable request directed to the corresponding author (HE). Full transcripts are not publicly available due to patient confidentially.
